# Reliability and validity of knee extensor strength measurements using a portable dynamometer anchoring system in a supine position

**DOI:** 10.1186/s12891-019-2703-0

**Published:** 2019-07-08

**Authors:** Kwan-Sik Sung, You Gyoung Yi, Hyung-Ik Shin

**Affiliations:** 1 Department of Rehabilitation Medicine, Seoul National University Hospital, Seoul, Republic of Korea; 2 Department of Rehabilitation Medicine, Seoul National University Hospital, Seoul National University College of Medicine, 101 Daehak-Ro, Jongno-Gu, Seoul, 03080 Republic of Korea

**Keywords:** Hand-held dynamometry, Supine, Portable dynamometer anchoring system

## Abstract

**Background:**

Muscle strength measurements using hand-held dynamometry (HHD) can be affected by the inadequate strength of the tester and lack of stabilization of the participants and the device. A portable HHD anchoring system was designed that enabled the measurement of isometric knee extensor muscle strength in a supine position. This can be used with individuals who are unable to assume the sitting position required for the measurement of knee extensor strength in conventional isokinetic dynamometry (IKD). The aim of this study was to evaluate the reliability and validity of knee extensor strength measurements using this device.

**Methods:**

The maximal knee extensor isometric strength of the dominant leg in healthy adults aged 20 to 40 years was tested. Three trials of three contractions were assessed by two raters using the portable dynamometer anchoring system whilst the participant was in the supine position. After the three measurement trials, peak knee extensor torque was evaluated using IKD. The intraclass correlation coefficient (ICC) and 95% limits of agreement (LOA) for intra- and inter-rater reliability were obtained.

**Results:**

Thirty-nine participants (19 male and 20 female, aged 30.08 ± 4.16 y), completed the three measurement trials. The ICC for intra-rater reliability was 0.98 for the maximum measurements of knee extensor strength (95% confidence interval [CI]: 0.96–0.98) and 0.98 (95% CI: 0.96–0.99) for inter-rater reliability. The mean difference (%) between the maximum knee extensor strength measurements of each trial was 1.02% (LOA range: − 11.13 to 13.16%) for intra-rater and − 1.44% (LOA range: − 13.98 to 11.08%) for inter-rater measurements. The Pearson correlation coefficient of the maximum voluntary peak torque measurements with the portable dynamometer anchoring system and IKD was 0.927.

**Conclusions:**

The portable dynamometer anchoring system is a reliable and valid tool for measuring isometric knee extensor strength in a supine position. Future clinical feasibility studies are needed to determine if this equipment can be applied to people with severe illness or disabilities.

**Trial registration:**

KCT0003041.

**Electronic supplementary material:**

The online version of this article (10.1186/s12891-019-2703-0) contains supplementary material, which is available to authorized users.

## Background

Loss of muscle strength in adulthood has been linked to frailty [1], increased risk of disability [2, 3], and mortality [4]. Lower extremity muscle strength affects postural stability and gait [5] and also predicts survival in middle age and later life [6, 7]. Therefore, lower-extremity muscle strength assessment is essential for clinicians [8].

Muscle strength can be evaluated using manual muscle testing (MMT), hand-held dynamometry (HHD), and isokinetic dynamometry (IKD) [9]. Although IKD is considered to be the gold standard for measuring strength [10], the equipment is large, expensive, lacks portability, and requires time-consuming testing and training sessions [11–13]. Thus, the application of IKD is impractical in many clinical settings.

Compared to IKD for muscle strength assessment, HHD devices are simple, portable, relatively inexpensive, and can be used at the bedside [11, 12, 14]. However, the accuracy of HHD measurements can be affected by lack of stabilization of the participants and the device [9]. Especially, isometric strength can be underestimated if the examiner is unable to oppose enough force to keep the tested limb in a fixed position [15].

To address the concerns associated with HHD devices, several anchoring systems to fix the HHD have been used with favorable results [10, 16–20]. However, these anchoring systems required the HHD to be fixed to the wall [10, 16] or to be constructed on the examination table prior to use [17–21]. When the system is used on the patients’ bed, the bed must be moved to an appropriate place for the measurement, or the anchoring systems must be installed every time another patient is measured. Therefore, it may be more practical to move patients to the laboratory rather than to conduct bedside measurements. However, this is not possible in severe cases such as in patients who are admitted to the intensive care unit. Therefore, although portability is considered to be an advantage of HHD, the HHD anchoring systems reported in previous studies limit portability.

We designed a simple, more portable HHD anchoring device that can measure knee extensor muscle strength in a supine posture on a hospital bed. The aim of this study was to examine the reliability and validity of knee extensor strength measurements using this anchoring system. It was hypothesized that the HHD anchoring system would demonstrate good intra- and inter-rater reliability for knee extensor strength measurement (< 15% limit of agreement).

## Methods

### Subjects

Healthy adults, aged 20 to 40 years, were enrolled in the study. Participants with a history of traumatic spine or lower extremity injury within the past six months, or who were unable to give written consent or understand the procedures of the experiment were excluded. The Seoul National University Hospital Institutional Review Board (No. 1801–072-916) approved the study and written informed consent forms were obtained. The participants were fully informed of the study purpose and procedures prior to enrollment. Analysis of pilot study data using the 95% limits of agreement (LOA) confidence interval, indicated that a minimum of 31 participants were required to demonstrate good intra- and inter-rater reliability (< 15% LOA). The target number of participants was determined through pilot data analysis (Additional file 1).

### Portable dynamometer anchoring system

**Fig. 1:**
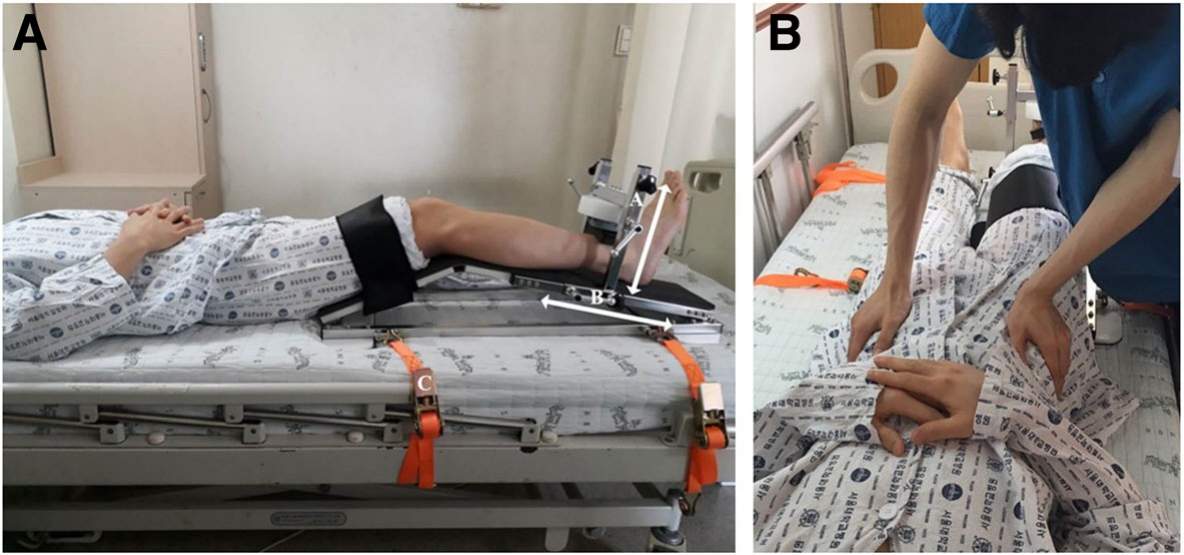
The portable dynamometer anchoring system. (**a**) The portable dynamometer anchoring system for measuring supine isometric knee extensor strength. A: Movable frame that can adjust the placement of the hand-held dynamometer (HHD) according to the size of the lower leg. B: Movable frame that can adjust the HHD placement according to leg length. C: Belt to fix the anchoring system to the bed. (**b**) Position of the examiner to prevent pelvic rotation of the participant

The knee flexion angle was fixed at 35° on the portable anchoring system (Fig. 1A). At this angle, higher knee extensor EMG activity [22] and favorable measurement reliability has been demonstrated [23, 24]. The frame, which was positioned perpendicular to the tibia, was designed to be moved up and down depending on the size of the leg. The HHD was placed 5 cm proximal to the lateral malleolus. The position of the HHD could be adjusted according to leg length.

The base of the anchoring system has four ‘U’ shaped rings, which allow the instrument to be fixed to the hospital bed. The thigh of each participant was fixed using a Velcro strap to minimize the compensatory action of hip flexion. A battery-operated, microFET II TM load cell system (Hoggan health industries, Draper, UT, USA), with a digital display of peak force ranging from 12.1 N to 1334.5 N, in 0.4 N increments, was anchored to the device.

### Measurement using the anchoring system

Isometric knee extensor strength (N) was measured using the portable dynamometer anchoring system in a supine position. Before the measurements, the position of the HHD was adjusted according to leg length and thickness. Each participant completed a familiarization session that included three knee extensor contractions of the dominant leg, defined as the preferred leg for kicking. The participants’ arms were positioned loosely across their chest. The assessment consisted of a total of three trials, with three maximal isometric contractions per 5 s of each trial. Thus, a total of nine contractions of the dominant leg knee extensors were performed by each participant. The maximal force values (N) from the three trials were used for analysis. The measurement commenced following a ‘kick’ sound, which was recorded by rater 2 in advance [20]. When the participants forcefully extended their knees, the examiner exerted pressure on the anterior superior iliac spine so that participants could not compensate by pelvic rotation (Fig. 1B). The first and second trials were evaluated by rater 1, and the third was evaluated by rater 2. The rest interval between trials was 30 min, and the rest interval between contractions was 30 s. To minimize fatigue in patients who cannot be in a sitting position, the rest interval was longer than the 5 to 10 min used in previous studies with normal subjects [16, 24, 25]. However, it was shorter than the one hour of rest interval employed in hematologic malignancy patients [26]. The lever arm length (m), from the knee joint to the HHD (5 cm proximal to the lateral malleolus), was measured by rater 1. The participants were instructed to inform the examiner if they experienced any pain or general discomfort during the testing procedure. They were also informed that the testing procedure could be stopped at any time upon request.

### Measurement using an isokinetic dynamometer

Isometric knee extension strength (Nm) was measured using a Biodex system 4 pro (Biodex Medical Systems Inc., Shirley, New York). Participants were seated with an 85° hip flexion angle and a 90° knee flexion angle [27], which is a standard method of measuring knee extensor strength in IKD. To assess the validity of the portable dynamometer anchoring system developed, we tried to analyze the correlation between torque values obtained using the anchoring system and IKD rather than compare the absolute torque values produced by both devices. The chest and pelvis were secured to the chair using Velcro straps, and a padded ankle strap was placed 5 cm proximal to the lateral malleolus. The isokinetic dynamometer was interfaced with an external data acquisition system (MP150; Biopac Systems, Inc.; Goleta, CA). Participants performed three maximal isometric contractions of 5 s with a 30 s rest between each contraction 30 min after the HHD measurements.

### Statistical analyses

A two-way random effect model (intra-class correlation [ICC] _2.1_), was used to examine intra- and inter-rater reliability. ICCs > .75 were deemed to represent good reliability, .50 to .75 moderate reliability, and < .50 poor reliability [28]. The correlation between the HHD measurement values (N) multiplied by leg length (m) and the torque values (Nm) measured with the Biodex system was analyzed using Pearson correlational analysis.

The standard error of measurement (SEM) was calculated using the following formula: SEM = SD (√1-ICC), where SD represents the standard deviation [29]. The minimal detectable change (MDC) was calculated as 1.96xSEMx√2 [29]. To calculate the LOA, the mean **±** (t_0**.5**, d.f. n-1_) (s _diff_) √1+ 1/n was used [30]. In the Bland and Altman plots, the differences were expressed both as absolute values (N) and percentages (%). The latter were calculated using the method described by Giavarina [31].

A repeated measures ANOVA was carried out using the three maximal isometric knee extensor strength measurements from each trial to test for learning or fatigue effects [32]. All statistical analyses were performed using the statistical package for the social sciences (SPSS) for windows (version 23, SPSS, IBM Corporation, New York, NY, USA).

## Results

Forty healthy participants (20 males, 20 females) with a mean ± standard deviation (SD) age of 30.1 ± 4.2 y, height of 169.8 ± 7.2 cm, and body mass of 65.4 ± 13.6 kg were enrolled in the study. One participant dropped out due to knee pain after the first session. The repeated measures ANOVA yielded no significant differences (*p* = 0.059) between the three maximal knee extensor strength measurement trials. This indicated that there were no learning or fatigue effects between the first and third trials.

### Intra-rater and inter-rater reliability

The mean maximal isometric knee extensor strength values measured in each trial using the portable dynamometer anchoring system in healthy adults (*n* = 39) are presented in Table 1. The intra- and inter-rater reliability for the maximal knee extensor strength measurements in each trial are presented in Table 2.

**Table 1:** Maximal isometric knee extensor strength measurements for each trial and each rater

Measurement	Mean (N)	SD (N)	Range (N)
Trial 1 – rater 1	492.00	157.1	213.3–813.8
Trial 2 – rater 1	486.87	153.0	239.3–761.2
Trial 3 – rater 2	499.32	159.2	238.0–799.0

SD: standard deviation; N: Newtons

**Table 2:** Intra- and inter-rater reliability of maximal isometric knee extensor strength in each trial

	ICC Mean (95% CI)	SEM (%) Mean (SD)	MDC (%) Mean (SD)
Intra-rater	0.98 (0.96–0.98)	21.8 (4.5)	60.4 (12.3)
Inter-rater	0.98 (0.96–0.99)	22.2 (4.5)	61.6 (12.4)

ICC: intraclass correlation coefficient, CI: confidence interval, SEM: standard error of measurement, MDC: minimal detectable change

Both the intra and inter-rater comparisons showed an excellent level of reliability. The MDCs for the intra- and inter-rater measurements were 60.39 N (12.34%) and 61.58 N (12.42%), respectively.

**Fig. 2:**
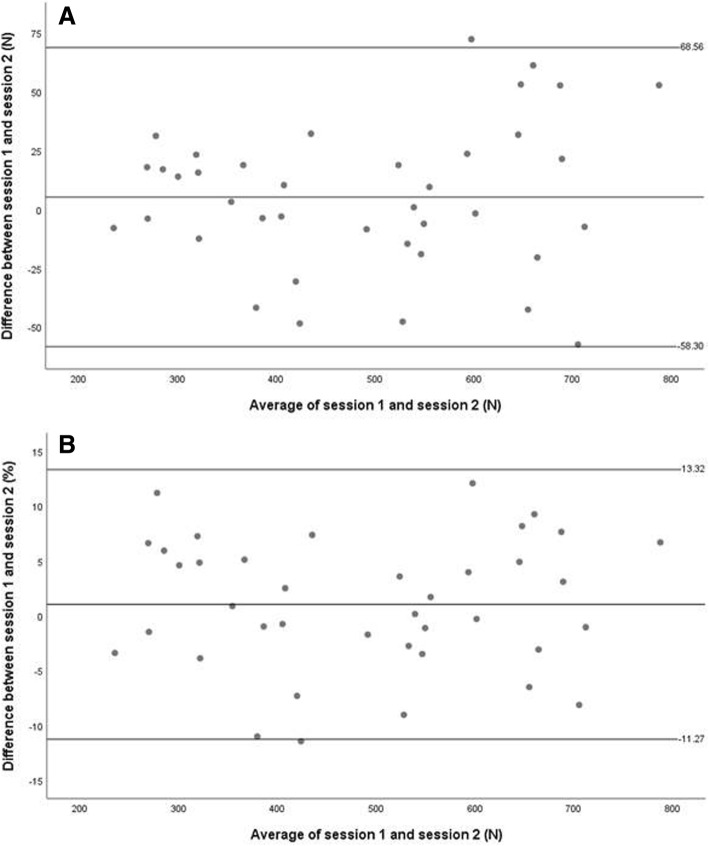
Bland and Altman plot for intra-rater measurements. (**a**) The mean difference and limits of agreement (LOA) between the maximum knee extensor strength measurements, (**b**) The mean difference (%) and LOA (%) between the maximum knee extensor strength measurements

For the intra-rater measurements, the average difference between the two isometric knee extensor strength measurement trials (Fig. 2A) was 5.13 N (LOA range: − 58.30 to 68.57 N). The average difference (%) between the two trials (Fig. 2B) was 1.02%, with an LOA range from − 11.28 to 13.32% [31].

**Fig. 3:**
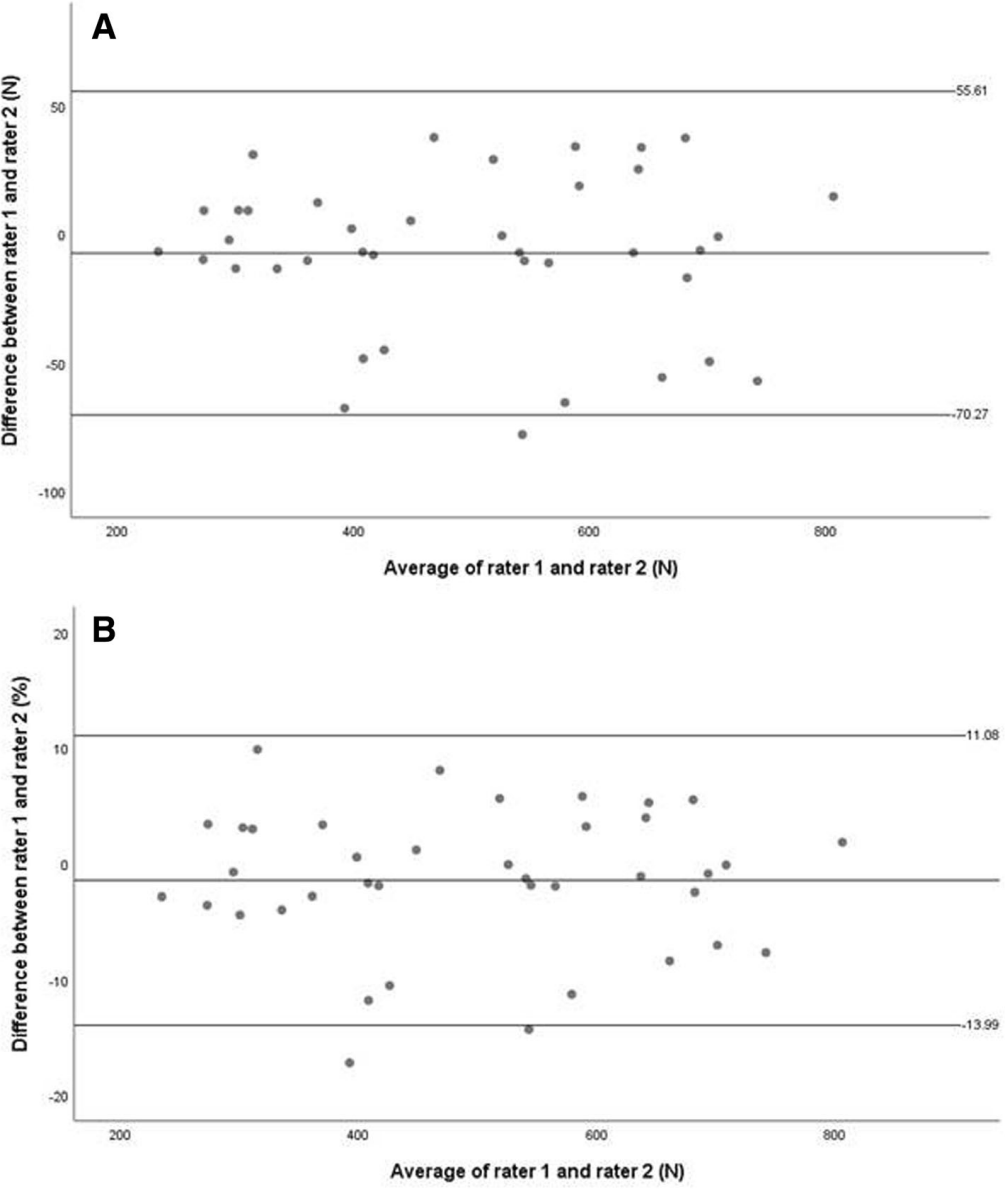
Bland and Altman plot for inter-rater measurements. (**a**) The mean difference and limits of agreement (LOA) between the maximum knee extensor strength measurements, (**b**) The mean difference (%) and LOA (%) between the maximum knee extensor strength measurements

For the inter-rater isometric knee extensor strength measurements, the average difference between the first and the second rater (Fig. 3A) was − 7.33 N (LOA range: − 70.27 to 55.61 N). The average difference (%) between the first and the second rater (Fig. 3B) was − 1.45% with an LOA range from − 13.99 to 11.08%.

### Validity of the portable dynamometer anchoring system measurements

**Fig. 4:**
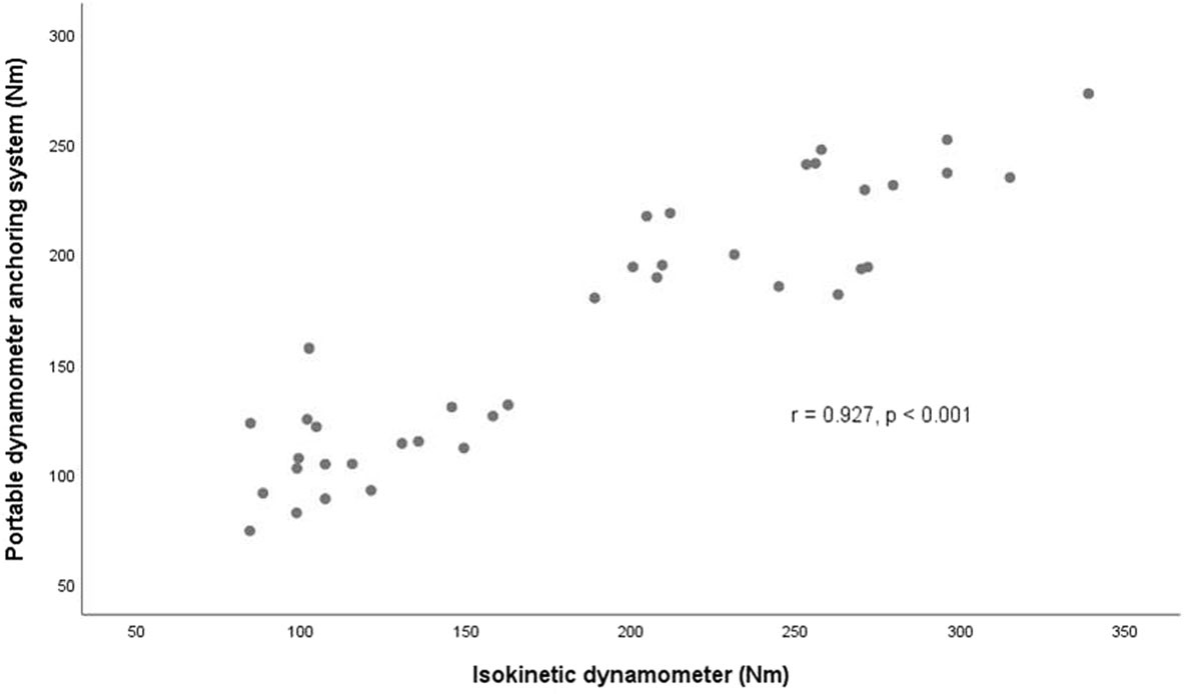
Correlation between maximal voluntary knee extensor torque obtained using the portable anchoring system and isokinetic dynamometer. r: Pearson correlation coefficient

The mean peak torque values obtained using the portable dynamometer anchoring system and IKD (Biodex) were 165.0 **±** 58.7 Nm and 186.1 **±** 77.5 Nm, respectively. The ICC was 0.85 for the maximum measurements of knee extensor strength (95% CI: 0.61–0.93). There was a significant correlation between the maximal voluntary peak torque from the portable anchoring system (obtained by multiplying the force by the lever arm length) and the values generated from the isokinetic dynamometer (r = 0.927, *p* < 0.001) (Fig. 4).

## Discussion

The dynamometer anchoring system developed in this study produced excellent intra- and inter-rater reliability for maximal isometric knee extensor strength measurements. These results are similar to the findings of other HHD fixation studies [10, 21]. Jackson et al. reported an intra-rater ICC of 0.93 for isometric knee extensor strength using a portable polyvinyl chloride pipe stabilization device [10]. Koblbauer et al. reported excellent ICCs for intra- and inter-rater reliability of knee extensor muscle strength measurements (0.92–0.97 and 0.95–0.96, respectively), when the HHD was fixed to a frame on a table using straps [21].

The advantage of the system developed in this study is that it can be applied to patients who are unable to walk or assume a sitting position. Therefore, unlike in previous studies, patients can be examined whist they are lying in a bed. Previous studies used anchoring systems that required the patient to be examined in a sitting position or moved to a laboratory. Due to the difficulty of directly measuring muscle strength in patients in intensive care units, ultrasonography has been used as a surrogate measurement technique to identify future impairment and changes in muscle strength and function [33–35]. However, the HHD anchoring system developed in this study could be used in this setting to directly measure muscle strength and to predict future body function. Further advantages of the portable dynamometer anchoring system are that it weighs 10.2 kg and can either be carried or transported in small carts. It is quick to install on a bed (approximately 10 min), and the examination time is less than 5 min (including the time taken to explain the procedures to the participants).

The limitation of the anchoring system developed in this study is that it only measures knee extensor strength and it cannot be used to measure the strength of other muscle groups including the hip abductors and hip extensors. However, knee extensor strength is an important determinant of human locomotor efficiency and stability [36–39] and it is positively associated with physical activity level and quality of life [40, 41].

We presented the MDC to examine the minimal amount of change that is required to distinguish a true performance change from a change due to variability in performance or measurement error when applying this anchoring system to healthy subjects. The MDC was 60.39 N (12.34%) for the intra-rater comparisons and 61.58 N (12.42%) for the inter-rater comparisons. A few studies have assessed the reliability of IKD. In the study by Mentiplay et al., the MDC for inter-rater reliability using IKD (KinCom) in healthy adults was 15.72% [25]. Another IKD study reported an MDC of 17.73% in patients with osteoarthritis [42]. Using Biodex IKD, Adsuar et al. found the MDC to be 21.5% in patients with fibromyalgia [43]. The MDC values obtained for the HHD anchoring system developed in this study are lower than those reported in previous studies that used IKD. The supine position could have contributed to these results because the body contact area was greater than in the sitting position, suggesting the possibility of better stability in the supine position during muscle strength measurement.

The LOAs presented in this study (− 11.13 to 13.16% for intra-rater, and − 13.98 to 11.08% for inter-rater measurements), were also comparable to those presented in previous studies using IKD. When examining knee extensor torques in children using a Biodex IKD, Tsiros et al. reported the LOA range to be − 41.3 to 21.8 Nm (− 30.19 to 15.94%) [44]. Adsuar et al. and Kean et al. also investigated knee extensor strength using an IKD, with the LOAs ranging from < − 15% to > 17% [42, 43].

There are a few limitations of this study. Firstly, a learning effect may have occurred when exerting knee extension force using the anchoring system developed in this study. Although a significant learning effect was not demonstrated by the repeated measure ANOVA (*p* = 0.059), the mean force value for session 3 was 499.33 N, which was higher than that of session 1 (492.00 N) and session 2 (486.87 N). Although there was one training session for familiarization before the measurement trials, more training sessions could be required in future studies. Secondly, the participants were healthy volunteers. The knee extensor strength measurement method proposed in this study is advantageous, since it can be used with severely ill or disabled persons who cannot assume a sitting posture or move to a laboratory. Future studies examining the efficacy of this portable anchoring system with specific populations or in environments such as intensive care units are needed.

## Conclusions

Measurements of isometric knee extensor strength in a supine position using the portable dynamometer anchoring system designed for this study showed a high level of reliability and validity in healthy subjects. Future clinical feasibility studies are needed to determine if this equipment can be applied to people with severe illness or disabilities.

## Additional file

Additional file 1: Pilot data analysis results. (DOCX 25 kb)

## Data Availability

Data is available upon reasonable request from the corresponding author.

## Abbreviations

CI: Confidence interval
HHD: Hand-held dynamometry
ICC: Intraclass correlation coefficient
IKD: Isokinetic dynamometer
LOA: Limits of agreement
MDC: Minimal detectable change
MMT: Manual muscle test
SD: Standard deviation
SEM: Standard error of measurement
