# Supplementary material for: Reliability and validity of knee extensor strength measurements using a portable dynamometer anchoring system in a supine position
Source: BMC Musculoskelet Disord. 2019 Jul 8;20:320. doi: 10.1186/s12891-019-2703-0 (PMC6615264; doi:10.1186/s12891-019-2703-0)
Supplement: Supplementary file 1 — Pilot data analysis results. (DOCX 25 kb) [file 12891_2019_2703_MOESM1_ESM.docx]

**Additional file 1.** Pilot data analysis results

Since the difference between the two measurements is expected to increase as muscle strength increases, the difference relative to the average of the two measurements is used. The measured value from each rater is termed as HDD_H and HDD_S. The difference between the two measured values ​​= ((HDD_H-HDD_S) / average) * 100

The distribution of the differences between the two measurements relative to the average is as follows:

|  | Average | Standard deviation | Median value | Minimal value | Maximal value |
| --- | --- | --- | --- | --- | --- |
| Difference between the two measured values (%)^1^ | 0.81 | 5.07 | 0.78 | -6.93 | 9.24 |

^1^: = ((HDD_H- HDD_S)/ Average)*100


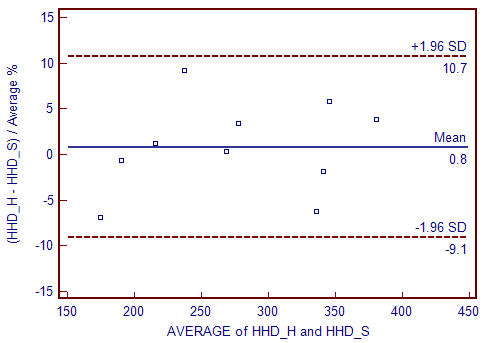


The upper/lower limit for 95% limits of agreement (LOA) was determined so that there was an 80% chance that the single-sided confidence interval would be clinically meaningful (= δ) 15%.

$$n=\frac{\sigma^{2}{(\sqrt{3}z_{1-\alpha}+\sqrt{2}z_{1-\beta})}^{2}}{{{(\delta-2\sigma)}^{2}}}$$

The n from the above equation is estimated to be less than the actual n. Therefore, the corrected LOA confidence interval was set to 1-β = 0.95 in order for the probability that it exists within δ to be 80%. $z_{1-\alpha}=1.645,z_{1-\beta}=1.645, \sigma=5.1\%, \delta=15\%$

Therefore, a minimum of 31 participants were required. If the dropout rate is estimated to be 20%, 40 participants are required.
